# Evaluation of extracts from *Sida acuta, Phyllanthus amarus, Parkia biglobosa* and their herbal ointment for therapeutic and biological activities

**DOI:** 10.1016/j.heliyon.2023.e19316

**Published:** 2023-08-19

**Authors:** Addai-Mensah Donkor, Benjamin Ahenkorah, Timothy Ajigepungu Wallah, Abdallah Yakubu

**Affiliations:** a Department of Pharmaceutics, School of Pharmacy and Pharmaceutical Sciences, University for Development Studies, Tamale, Ghana; b Department of Medical Laboratory Science, Bolgatanga Technical University, Bolgatanga, Upper East Region, Ghana; c Department of Applied Chemistry, University for Development Studies, Tamale, Ghana; d Department of Pharmaceutical Chemistry, School of Pharmacy and Pharmaceutical Sciences, University for Development Studies, Tamale, Ghana

**Keywords:** *Escherichia coli*, *Staphylococcus aureus*, *Parkia biglobosa*, *Sida acuta*, Polyethylene glycol (PEG), *Pseudomonas aeruginosa*

## Abstract

Herbal extracts are a well-known source of therapeutically important bioactive chemicals since they are widely available, relatively inexpensive, and have fewer adverse effects. The three plants' leaves have been used to treat a variety of illnesses in Ghana, including skin conditions and wound infections. Their effectiveness as an ointment in treating the aforementioned illnesses has not yet been shown, though.

The extracts were made into an ointment with polyethylene glycol (PEG), and both the ointment and the raw extracts were examined for in vitro antibacterial activity. The three (3) chosen bacterial isolates were subjected to potential activities of the plant extracts from different extractants.

The minimum bactericidal concentration (MBC) and minimum inhibitory concentration (MIC) values for the plant extracts were both low. The herbal ointment made with *Sida acuta* extract from both extractants showed significantly different activity (P < 0.05), against the test pathogens when compared to the reference medication (Madecassol®). However, the activities of formulated herbal ointment from both *P. amarus* and *P. biglobosa* extracts were comparable at higher concentrations to the standard drug used. Notably, both plant extracts and extract-PEG manufactured ointments exhibit significant in vitro efficacy against the disease-causing bacterial species.

The current study is the first in-depth account of *Parkia* species with regard to an examination of herbal ointments made from leaves extract obtained utilizing solvents such as water and ethanol. Our research findings have important implications for the pharmaceutical industry in terms of providing a suitable, workable, and alternative supply of bioactive compounds and anti-infective agents.

## 1 Background

Indigenous medicinal plants were present and still hold potential as sources of modern medications [[1], [2], [3], [4], [5], [6]]. Significantly, the popularity of using phytotherapy as an alternative form of treatment has increased interest in the pharmacognosy of tropical plants [2,7]. A huge portion of the population in Africa, particularly in Ghana, benefits greatly from the use of medicinal plants in healthcare. Mostly because they are affordable, easily accessible and effective locally. It has been reported that *Sida acuta's* various parts have been used to treat a range of ailments, including neurological disorders, headaches, abnormal vaginal discharge, tuberculosis, diabetes, malaria, and other fevers, uterine disorders, rheumatic disorders, renal inflammation, asthma, ulcers, and infections related to childbirth [[8], [9], [10], [11]]. By examining the pain brought on by a few particular disorders, Konaté et al.'s ethnobotanical research [12] discovered that various plant species have historically been utilized to treat a variety of pain types. The most common and commonly used of these species are *S. acuta Burne f*. and *S. cordifolia* L. (Malvaceae). In most cases, these plants are administered over a long period of time and their dosage is not strictly regulated [2,5,11,[13], [14], [15]]. The efficacy of these herbs in the treatment of infectious diseases is well established. Traditional medicine has used the herb, *Phyllanthus amarus*, to treat a variety of illnesses, including diarrhea, dysentery, dropsy, jaundice, intermittent fevers, urogenital diseases, scabies, and wounds [9,16]. The plant is also used to treat chronic diarrhea, gonorrhea, intraluminal bladder pathologies, pain, and diabetes [[17], [18], [19], [20], [21]]. It is applied topically to treat a variety of skin conditions, including ringworm, scabies, skin ulcers, sores, swelling, and itching, wounds, bruises, and scabies [18,20,22,23]. The plant is also used to treat prostate diseases, appendiceal mass and abscess, and renal calculi/gallstones [21,24].

According to research, *Parkia* species are utilized in the majority of tropical nations to treat a variety of diseases [[25], [26], [27]]. African locust bean tree, also known as *Parkia biglobosa*, is a multifunctional plant that is extensively used by indigenous populations in dry Africa. The local population is familiar with the plant and uses it in a variety of ways, and local knowledge of the species is diverse and influenced by several ethnic groups. Unsurprisingly, racial inequalities in the species' use values and usage behaviors were found by Koura et al. [28]. The leaves, pods, and roots of *P. biglobosa* have traditionally been used to treat diabetes mellitus, skin conditions like eczema, skin ulcers, measles, leprosy, wounds, dermatitis, chickenpox, scabies, and ringworm, as well as other ailments [26,27,29,30]. According to studies [26,29], the stem barks and leaves of *P. biglobosa* are administered as paste and decoction to cure various skin-related disorders. Hypertension has also been treated using decoction and paste made from *P. biglobosa* stem bark and pod [31,32]. Additionally, the leaves and stem bark of *P. biglobosa* are used to treat bronchitis and severe coughs [33]. Based on the aforementioned applications, it is believed that *Parkia* plants contain components with a wide range of biological activities, including antidiabetic, antibacterial, antihypertensive, and anti-inflammatory effects. Although, the leaves of the above-mentioned plants have been thoroughly explored in several pathologies, however, there is a research gap in the potential of the herbal extract ointment of the leaves for the treatment of skin related infections and in wound healing. Hence, the current research aimed at formulating ointments of extracts obtained from different extractants of the leaves of *Sida acuta, Phyllanthus amarus* and *Parkia biglobosa* found in Ghana with the aid of polyethylene glycol (PEG), thus, evaluating the crude extracts and the extract-PEG ointment on skin and wound infections causally related to infectious pathogenic bacteria. The study draws its major contribution from establishing a promising anti-infective potency of the crude extracts and the extract-PEG ointments on skin and wound pathogenic bacteria isolates.

## 2 Methods

### 2.1 Collection of plant materials

**Fig. 1:**
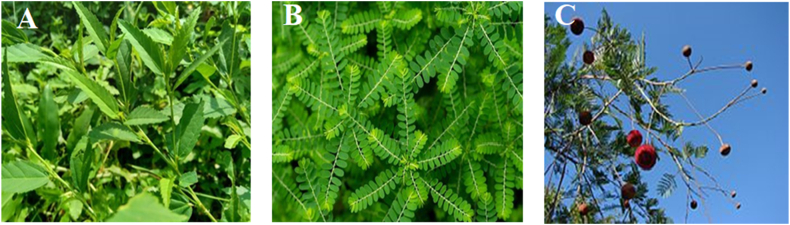
Shows leaves of A: *Sida acuta*, B: *Phyllanthus amarus* and C: *Parkia biglobosa.*

Plant components from *Sida acuta, Phyllanthus amarus*, and *Parkia biglobosa* were harvested in Navrongo, Ghana's Upper East Region, between August and September 2021. Professor Isaac Sackey, a plant taxonomist in the Department of Applied Biology at the University for Development Studies, Tamale, observed and verified the plants (Fig. 1).

Fig. 1 shows the leaves of the three (3) plants used to prepare the crude extracts.

### 2.2 Preparation of plant crude extracts

*Sida acuta, Phyllanthus amarus*, and *Parkia biglobosa* leaves were rinsed with distilled water and dried for four weeks in the shade to ensure the leaves were fully devoid of moisture. Using a mortar and pestle, the dried leaves were pounded into a uniform powder and stored in a fresh, airtight container. Powdered leaf samples (1.5 kg) were soaked for 72 h at room temperature in 5 L of each extractant to create the ethanol and aqueous extracts. In order to optimize the extraction of the bioactive phytochemicals, the solutions were sometimes shaken. Whatman filter paper No. 42 was used to separate the extracts, and they were then concentrated using a rotary evaporator (Heidolph 4001 Laborota Rotary Evaporator), which was warmed on a water bath at 70 °C for the aqueous extract and 50 °C for the ethanol extract to produce semi-solid products. Percentage weigh/weight (% w/w) calculations were used to determine the yields.

### 2.3 Phytochemical screening

To identify the existence of bioactive components, phytochemical screening of the plant extracts was carried out using the technique described by Donkor et al. with a few modifications [34].

### 2.4 Formulation of an ointment with crude extract and polyethylene glycol (PEG)

Following the procedure outlined by Donkor et al. [35], 30 g of polyethylene glycol 400 (PEG 400) and 4000 (PEG 4000) were each weighed into a beaker and heated at 45 °C until liquefied. After that, the liquid was congealed by stirring it with a glass rod submerged in room temperature tap water. PEG ointment was used to formulate individual leaf extracts. Each extract was weighed into clean, labeled beakers in the amounts of 250 mg, 125 mg, and 62.5 mg. One gram (1.0 g) of the PEG ointment was then added to each beaker and heated for approximately 30 min at 70 °C while being regularly stirred. The mixtures were allowed to cool at room temperature in order to create an extract-PEG ointment with various concentrations of 250 mg/g, 125 mg/g, and 62.5 mg/g. The reference medication was 0.5 g of Madecassol (Bayer, 00001199), which includes 1% of a Centalla asiatica extract known for cicatrization (wound healing).

### 2.5 Quality control parameters of formulation

The pH, spreadability, and extrudability tests were carried out as quality control procedures on the formulation at various concentrations. The pHs of the different formulations were tested three times with a digital pH meter. Briefly, 200 ml of distilled water were used to dissolve 1 g of the herbal ointment before it was kept for 3 h. The ointment was applied between two slides under the control of a certain load, and the spreadability was measured in terms of how long it took the slides to separate from the ointment (in seconds). The weight (g) necessary to extrude a 0.5 cm ribbon of ointment in 5 s was used to determine the extrudability of the ointment formulations. Physical characteristics of the herbal ointment compositions, such as color, odor, smoothness, and grittiness, were also noted.

### 2.6 Test microorganisms

This study made use of clinical isolates of *P. aeruginosa, E. coli*, and *S. aureus*. The Microbiology Unit of the Tamale Teaching Hospital in the Northern Region of Ghana, provided the isolates. Morphological and biochemical methods were used to validate the test organisms' identities [36,37]. In nutrient broths, bacteria were kept alive between 2 and 8 °C.

#### 2.7 Agar well diffusion assay

For the agar well diffusion assay, Suurbaar et al.'s technique [38] was used.

### 2.8 Preparation of inoculum for minimum bactericidal concentration and minimum inhibitory concentration

Suurbaar et al.'s method was used to produce the cultures for the minimum inhibitory concentration (MIC) and minimum bactericidal concentration (MBC) tests [38] with only minor alterations. For the MIC and MBC experiments, a minimum of 3–5 pure colonies sub-cultured on an agar plate were employed. A sterile inoculating loop was used to transfer the inoculum into a tube containing 5 ml of ordinary saline, which was then vortexed. The inoculation broth that was produced as a result of this procedure was examined for approximately 4 h at 37 °C until it reached the turbidity of the 0.5 McFarland standards (1.5108 cfu).

### 2.9 MIC and MBC determination

MBCs were determined using the tube diffusion method, whereas MICs were determined using the agar well diffusion method [35].

### 2.10 Statistical analysis

The statistical analysis was carried out using Graphpad Prism 9. Zones of inhibition measured for the two sets of studies in each example were converted to means and standard deviation. To find a significant difference at P < 0.05, these means were statistically compared using the Two-Way Analysis of Variance (ANOVA) test. Dunnett's Post-hoc tests (multiple comparison test) were performed for variables which were confirmed to be significant after the ANOVA test. The test compared the means against the mean of the standard drug to see if there was a statistically significant difference. All tests were two-sided and p-value <0.05 was considered statistically significant difference.

## 3 Results

### 3.1 Antimicrobial studies of the crude extracts

The yield of each extract was expressed as a percentage (w/w) (Table 1). The highest yield was observed in aqueous extract of *Parkia biglobosa* (14.50% w/w) whiles ethanol extract of *Phyllanthus amarus* gave the lowest yield of (7.20% w/w).

**Table 1:** Plant extracts’ yield of respective extractants.

Plant Species	Extractant	Yield (% w/w)
*Parkia biglobosa*	Aqueous	14.50
	Ethanol	8.10
*Phyllanthus amarus*	Aqueous	12.40
	Ethanol	7.20
*Sida acuta*	Aqueous	9.30
	Ethanol	7.30

### 3.2 Profile of phytochemicals in plant extracts

The data obtained from the qualitative phytochemical analysis of the extracts from the three plants is shown in Table 2.

**Table 2:** Phytochemical profile of plant extracts.

Phytoconstituents	Extract					
	AESA	EESA	AEPA	EEPA	AEPB	EEPB
Tannins	+	+	+	+	+	+
Saponins	+	+	+	+	+	+
Polyuronides	+	+	+	+	**_**	**_**
Reducing sugars	+	+	+	+	**_**	**_**
Terpenoids	+	+	+	+	+	+
Flavonoids	+	+	+	+	+	+
Alkaloids	+	+	+	+	+	+
Anthraquinones	**_**	**_**	**_**	**_**	**_**	**_**

+ = **Detected; - = Not Detected**.

### 3.3 MICs and MBCs (mg/ml) of the herbal ointment and crude plant extracts against bacterial isolates

Low MIC values were obtained in the antimicrobial activity of the various plant extracts against the three (3) pathogens that were selected **(**Table 3**).** Because the test microorganisms were extremely sensitive to the extracts, it is clear that the extracts were efficacious against the microorganisms that were examined in this study.

**Table 3:** MICs and MBCs (mg/ml) of both crude plants extracts and the herbal ointment against the bacterial isolates.

Extract/Herbal Ointment		MIC						MBC					
		*E. coli*		*S. aureus*		*P. aeruginosa*		*E. coli*		*S. aureus*		*P. aeruginosa*	
a	a*	a	a*	a	a*	a	a*	a	a*	a	a*	a	a*
AESA	AESA-PEG	9.0	9.5	6.3	6.3	6.3	6.3	350	350	350	350	350	350
EESA	EESA-PEG	6.3	6.3	7.2	7.2	7.2	6.3	350	350	350	350	350	350
AEPA	AEPA-PEG	6.3	6.3	3.8	3.8	6.3	6.3	350	350	350	350	350	350
EEPA	EEPA-PEG	9.5	9.5	6.3	6.3	6.3	6.3	350	350	350	350	350	350
AEPB	AEPB-PEG	6.3	6.2	9.5	9.5	6.3	6.2	350	350	350	350	350	350
EEPB	EEPB-PEG	6.3	6.3	6.3	6.3	8.5	8.5	350	350	350	350	350	350

*AESA:* aqueous extract of *Sida acuta, EESA:* ethanol extract of *Sida acuta, EEPA:* ethanol extract of *Phyllanthus amarus, AEPA:* aqueous extract of *Phyllanthus amarus, EEPB:* ethanol extract of *Parkia biglobosa, AEPB:* aqueous extract of *Parkia biglobosa*, *P. aeruginosa*: *Pseudomonas aeruginosa, E. coli*: *Escherichia coli, S. aureus: Staphylococcus aureus,* MBC: minimum bactericidal concentration, MIC: minimum inhibitory concentration, a: raw extract, a*: raw extract-PEG ointment.

**Fig. 2:**
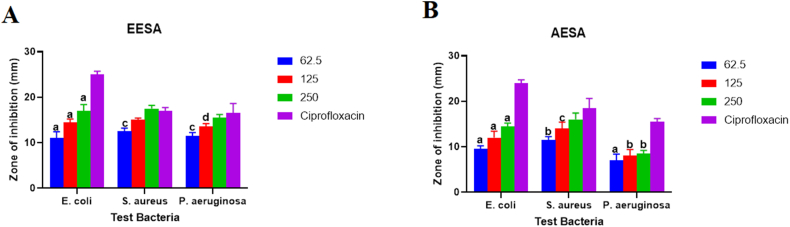
Shows (A) Antimicrobial activity of ethanol crude leaf extract, EESA (mm), and (B) Aqueous crude leaf extract, AESA (mm) of *S. acuta* plant. The data shows the crude extracts' diameter of inhibition against *E. coli*, *P. aeruginosa*, and *S. aureus*. On any given day, three wells on average were treated. The experiment was conducted twice, and it was discovered that daily variation was within one-fold of the data reported. a: P ≤ 0.0001, b: P ≤ 0.001, c: P ≤ 0.01, d: P ≤ 0.05 represent significant difference from the standard drug ciprofloxacin.

In terms of *Sida acuta* extract, the ethanol extract exhibited the largest diameter zone of inhibition against *S. aureus*, measuring 17.5 mm ± 1.41 mm, while the aqueous extract produced a diameter zone of inhibition of 16.0 mm ± 1.41 mm at the maximum concentration of 250 mg/ml (Fig. 2)*.* At the lowest concentration (62.5 mg/ml), *S. acuta* ethanol extract demonstrated the lowest inhibitory activity against both *E. coli* and *P. aeruginosa* with inhibition zones of 11.0 ± 0.71 and 11.5 ± 1.42 mm, respectively. At the highest dose of 250 mg/ml compared to the standard treatment, the *S. acuta* extract's activity against the test pathogens (*S. aureus* and *P. aeruginosa*) was not significantly different (P > 0.05). This suggests that the ethanolic extract's antimicrobial activities against *S. aureus and P. aeruginosa,* are comparable to the standard drug at the highest concentration, 250 mg/ml. However, there was a marked elevation (P < 0.05) in activity of the standard drug compared to all three concentrations of the ethanol extract against *E. coli* suggesting a limited activity of the three concentrations (62.5, 125 and 250 mg/ml) compared to the standard drug*.* The activity of the standard drug matched with the various aqueous extract variable concentrations was significantly different (P < 0.05) despite the fact that the aqueous extract did exhibit dose-dependent activity against the test microorganisms, with the exception of the 250 mg/ml dose against *S. aureus* (Fig. 2).

**Fig. 3:**
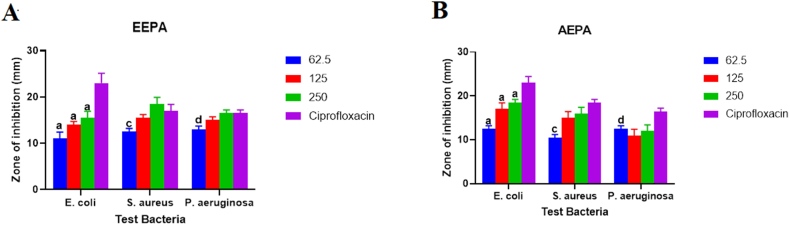
Shows (A) Antimicrobial activity of ethanol crude leaf extract, EEPA (mm), and (B) Aqueous crude leaf extract, AEPA (mm) of *P. amarus* plant. The data shows the crude extracts' diameter of inhibition against *P. aeruginosa*, *E. coli*, and *S. aureus*. On any given day, three wells on average were treated. The experiment was conducted twice, and it was discovered that daily variation was within one-fold of the data reported. a: P ≤ 0.0001, b: P ≤ 0.001, c: P ≤ 0.01, d: P ≤ 0.05 represent significant difference from the standard drug ciprofloxacin.

*Phyllanthus amarus* ethanolic crude extract had the highest diameter inhibition zone of 18.5 ± 2.12 mm at concentration of 250 mg/ml against *S. aureus,* whilst the aqueous extract had the highest diameter inhibition zone of 18.5 ± 0.71 mm at concentration 250 mg/ml against *E. coli* (Fig. 3). The experimental results further revealed dose dependency of the ethanol extract against all the test microorganisms but differences in activity were statistically insignificant (P > 0.05) at the 125 and 250 mg/ml doses for *S. aureus* and *P. aeruginosa*, suggesting similar antimicrobial activities of the extract compared to the standard drug at these higher concentrations. Interestingly, the aqueous extract exhibited similar antimicrobial activity at all concentrations tested against *P. aeruginosa,* comparable to the standard drug whilst it presented dose dependency inhibitory activity against both *E. coli* and *S. aureus* similar to the activity of the ethanolic crude extract on *E. coli and S. aureus* (Fig. 3). However, the three crude extract concentrations (both ethanolic and aqueous extracts) exhibited no inhibitory action against *E. coli* while the positive control (ciprofloxacin) against the bacteria utilized in this research significantly outperformed the three crude extract concentrations. DMSO - 99.99%, a negative control, however, displayed no inhibitory activity.

**Fig. 4:**
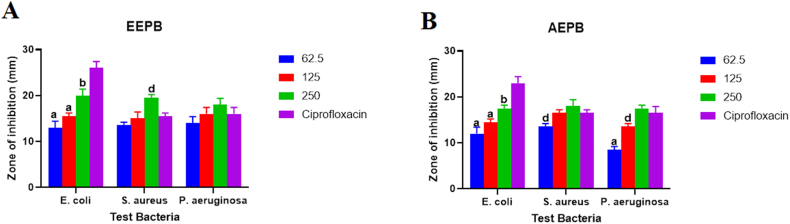
Shows (A) Antimicrobial activity of ethanol crude leaf extract, EEPB (mm), and (B) Aqueous crude leaf extract, AEPB (mm) of *P. biglobosa* plant. The data shows the crude extracts' diameter of inhibition against *P. aeruginosa*, *E. coli*, and *S. aureus*. The experiment was conducted twice, and it was discovered that daily variation was within one-fold of the data reported. a: P ≤ 0.0001, b: P ≤ 0.001, c: P ≤ 0.01, d: P ≤ 0.05 represent significant difference from the standard drug ciprofloxacin.

With regards to *Parkia biglobosa*, both ethanol and aqueous crude extracts generally showed dose dependence inhibitory activity against all the test microorganisms experimented. The standard drug had a higher inhibitory activity against *E. coli* (p < 0.05) compared to a relatively low inhibitory activity in relation to the other test bacteria used in this study (Fig. 4).

### 3.4 Antimicrobial studies of formulated *Extract-PEG ointment*

We used Polyethylene glycol (PEG) due to its biodegradable nature. PEG has been described as highly biocompatible and non-immunogenic**.** Its applications focus mainly on drug delivery and targeted diagnostics. It also shows outstanding toxicological safety in relation to acute and chronic oral toxicity, embryotoxicity and skin compatibility [39]. In cosmetics, food and the pharmaceutical industries, PEGs have been applicably listed for many years. An extra significant asset of PEG is the solvent power for numerous substances that are sparingly soluble in water [39].

**Fig. 5:**
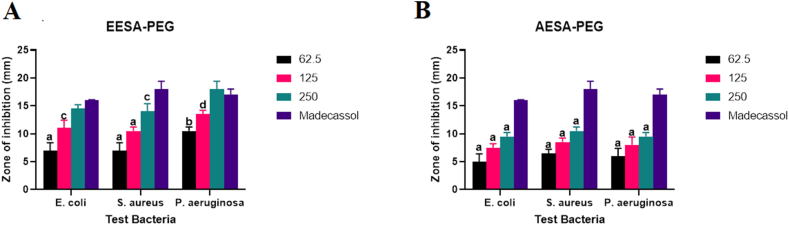
Shows (A) Antimicrobial activity of the formulated ethanol and (B) Aqueous extracts of *S. acuta*, EESA-PEG and AESA-PEG (mg/g), respectively. The data shows the ointment's diameter of inhibition against *P. aeruginosa*, *E. coli*, and *S. aureus*. On any given day, three wells on average were treated. The experiment was conducted twice, and it was discovered that daily variation was within one-fold of the data reported. a: P ≤ 0.0001, b: P ≤ 0.001, c: P ≤ 0.01, d: P ≤ 0.05 represent significant difference from the standard drug Madecassol.

Herbal ointment made from *S. acuta's* ethanolic extract, EESA-PEG Ointment, showed activity against the test pathogens, with *P. aeruginosa* experiencing the greatest inhibition at a zone with a diameter of 18 mm and a concentration of 250 mg/g (Fig. 5). In addition, the formulated ointment exhibited dose dependency activity against the pathogens as concentration was increased from 62.5 to 250 mg/g. Furthermore, the ointment was highly active at the highest concentration, 250 mg/g, as its antimicrobial activity at this concentration was similar to that of the reference drug as there was no significant difference between this high concentration and the standard drug against *E. coli* and *P. aeruginosa*. Although, the formulated herbal drug for the aqueous extract of *S. acuta*, AESA-PEG Ointment showed substantial dose dependence activity against all the test microorganisms, however its antimicrobial activity was markedly lower than that of the standard drug at the various concentrations against all the test microorganisms.

**Fig. 6:**
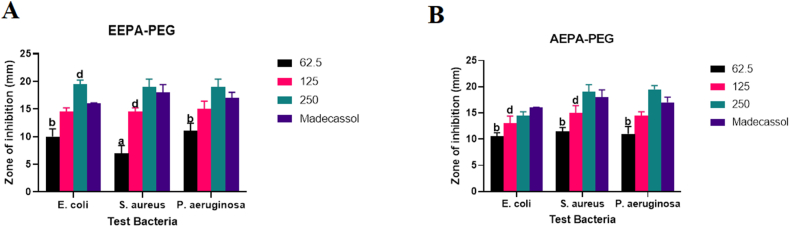
Shows (A) Antimicrobial activity of the formulated ethanol and (B) Aqueous extracts of *P. amarus*, EEPA-PEG and AEPA (mg/g), respectively. The data demonstrates the ointment's diameter of inhibition against *P. aeruginosa*, *E. coli*, and *S. aureus*. On any given day, three wells on average were treated. The experiment was conducted twice, and it was discovered that daily variation was within one-fold of the data reported. a: P ≤ 0.0001, b: P ≤ 0.001, c: P ≤ 0.01, d: P ≤ 0.05 represent significant difference from the standard drug Madecassol.

Formulated aqueous extract of *Phyllanthus amarus*, AEPA-PEG herbal ointment, exhibited potency against the microorganisms at all concentrations experimented with highest activity at a diameter inhibition zone of 19.5 ± 0.71 mm at a concentration of 250 mg/g against *P. aeruginosa.* The antimicrobial activity at this remarkably high concentration was higher than that of the reference drug (Fig. 6). Additionally, there was a dose dependency increase in activity against all test microorganisms. The formulated ethanol extract, EEPA-PEG ointment, inhibited the growth of the microorganisms in a dose dependence pattern with maximum inhibition at diameter zone of 19.5 ± 1.14 mm at a maximum concentration of 250 mg/g against *E. coli.* Furthermore, the antimicrobial activity at this remarkably high concentration was also higher than that of the reference drug.

**Fig. 7:**
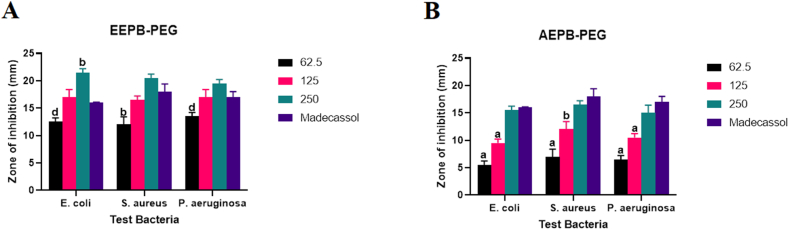
Shows (A) Antimicrobial activity of the formulated ethanol and (B) Aqueous extracts of *P. biglobosa*, EEPB-PEG and AEPB-PEG (mg/g), respectively. The data shows the ointment's diameter of inhibition against *P. aeruginosa*, *E. coli*, and *S. aureus*. On any given day, three wells on average were treated. The experiment was conducted twice, and it was discovered that daily variation was within one-fold of the data reported. a: P ≤ 0.0001, b: P ≤ 0.001, c: P ≤ 0.01, d: P ≤ 0.05 represent significant difference from the standard drug Madecassol.

The formulated ethanolic extract of *P. bigbolosa*, EEPB-PEG ointment, showed a dose dependence activity against all test microorganisms with the highest activity at an inhibition zone of 21 ± 0.71 mm corresponding to a concentration of 250 mg/g against *E. coli* (Fig. 7*)*. Additionally, both higher concentrations (125 and 250) mg/g, were comparable in activity with the reference drug, with the 250 mg/g concentration having a moderately elevated activity (P > 0.05) against *S. aureus* and *P. aeruginosa*, and a marked elevation in activity (P < 0.05) against *E. coli,* compared to the activity of the reference drug. At a concentration of 250 mg/g against *S. aureus*, the activity of AEPB-PEG ointment showed a high diameter zone of inhibition of 17 ± 1.41 mm and demonstrated a dose-dependent response against all test pathogens*.* Furthermore, the antimicrobial activity at the highest concentration of 250 mg/g was comparable to that of the reference drug.

## 4 Discussions

### 4.1 Antimicrobial studies of the crude extracts

The plants' leaves were utilized in the current study. *Parkia biglobosa* has historically been used to treat illnesses connected to wounds, diarrhea, and coughing. *Sida acuta* is used to cure conditions including ulcers, worms, diabetes, malaria, and other fevers, whereas *Phyllanthus amarus* is used to treat pathologies of the stomach, liver, and kidneys.

The findings of this study are consistent with a study report [40], that assessed the phytochemical and antibacterial efficacy of ethanolic and acetone leaf extracts on wound bacterial isolates and discovered that *S. acuta* had a broad spectrum antibacterial inhibitory effect against both Gram-positive and Gram-negative bacteria. Notably, the inhibitory effect was more prominent in Gram-positive bacteria than in Gram-negative bacteria, making the extract from *S. acuta* leaves the gold standard antibacterial medication for illnesses caused by Gram-positive bacteria. Further study to assess the effectiveness of the plant extract against viruses and fungus was suggested by the researchers.

Senthilkumar et al.'s research team discovered through phytochemical profiling of the *S. acuta* aqueous extract that the plant's antibacterial properties were linked to its phytoconstituents, including alkaloids, steroids, flavonoids, phenols, terpenoids, and cardiac glycosides. Additionally, they pointed out that at the maximum test dose of 60 μL, the aqueous plant extract had the highest zone of inhibition against *Bacillus subtilis* and *E. coli*.

Similar to the previous study, a greater variety of phytoconstituents, such as saponins, tannins, polyuronides, terpenoids, reducing sugars, flavonoids, and alkaloids, were examined in this study, which led to the discovery of the antibacterial properties of the plant's aqueous leaf extract. Additionally, studies on *S. acuta's* aqueous leaf extract have revealed that it has a moderate level of antibacterial activity against *S. aureus* and *P. aeruginosa* [41].

*M. smegmatis*, a soil-dwelling saprophyte related to *Mycobacterium tuberculosis*, was used in investigations on the mycobactericidal properties of *P. amarus* leaves [42,43]. Since employing the disease-causing tubercle bacillus directly has limitations, it is thought that adopting a mycobacterium model is useful in studying the pathophysiology of *M. tuberculosis* [42]. The researchers reported that *P. amarus* was active in both aqueous and 50% ethanol extracts, although the ethanol extract showed stronger activity as seen by a higher zone of inhibition. Thus, 50 mg/ml and 100 mg/ml were found to be the minimum inhibitory concentrations for the 50% ethanol and aqueous extracts, respectively. A more thorough investigation of the active 50% ethanol extract at 100 mg/ml in kill kinetic experiments revealed a significant decrease in the number of viable cells when compared with the untreated control at all stages throughout a 24-h time frame.

*Salmonella typhi* was used as the test organism in a different study to evaluate the antibacterial activity of ethanol and water extracts of *Phyllanthus amarus* [44]. *P. amarus* extracts in ethanol, cold water, and hot water were used to test their antibacterial properties. The ethanolic extract, with a diameter of 8.0 mm as a growth suppression zone, was shown to be the most effective against the test bacteria, according to the researchers. In another study, the antibacterial efficacy of *P. amarus* leaf extract against *P. aeruginosa, K. pneumonia, P. mirabilis, Enterobacter species, S. faecalis, S. aureus, Serratia marcescens,* and *E. coli* was examined in hexane, petroleum ether, chloroform, acetone, and methanol. The maximum inhibitory activity against the aforementioned bacterial species was found in the methanol extract of *P. amarus* [45].

The stem bark and leaves of *P. biglobosa* have been reported to show antibiotic properties [25,26,29,30]. Data on *P. biglobosa's* inhibitory activities are currently scarce. According to research reports on the inhibitory actions of ethanol extracts of both leaves and barks, *Salmonella* and *Shigella* isolates that were resistant to many drugs showed growth inhibition [46]. According to Saleh et al. bioactive components found in *Parkia* species, including phenolics, flavonoids, terpenoids, and volatile chemicals, may be responsible for the plant's wide range of medicinal characteristics and its numerous health benefits [47].

### 4.2 Antimicrobial studies of Extract-PEG ointment formulation

The herbal ointment made with *Sida acuta* extract from both extractants showed significantly different activity (P < 0.05) against the test pathogens when compared to the reference medication (Madecassol®). However, the activities of formulated herbal ointment from both *P. amarus* and *P. biglobosa* extracts were comparable at higher concentrations to the standard drug used. The quick evolution of antimicrobial medication resistance has accelerated the search for novel therapeutic options to treat illnesses brought on by bacteria and fungi. The in vitro activity of both the raw plant extracts and the extract-PEG ointment on the test organism used in this study is therefore highly noteworthy. However, further *in* vivo (laboratory animals) antimicrobial and toxicity studies on the test plants are warranted in order to extrapolate the findings in humans.

## 5 Conclusions

We have for the first time, successfully formulated extract-PEG ointments from the crude extracts obtained from the leaves of *Sida acuta*, *Phyllanthus amarus* and *Parkia biglobosa* plants found in Ghana. The anti-infective potency of the crude extracts and the extract-PEG ointments was evaluated on skin and wound infectious pathogenic bacteria isolates. The results of the study revealed a promising antibacterial inhibitory activity of the extract-PEG. Amongst the six extracts from the two extractants evaluated, the ethanol extract possessed the highest dose dependency inhibitory activity against all test organisms comparable to the standard drug used. Nonetheless, the bioactive compounds associated with this effect were not explored in detail hitherto. In order to assess the molecules linked to the inhibitory effects of the test plants after an improved and highly sensitive fractionation and purification procedure has been carried out, additional investigations concentrating on phytochemical profiling of these bioactive compounds are required. To determine the various plant extracts' safety margins, toxicological tests are also necessary. The ethnomedicinal properties of *Sida acuta*, *Phyllanthus amarus*, and *Parkia biglobosa* in Ghana are supported by scientific research in this study.

## Author contribution statement

Addai-Mensah Donkor: Conceived and designed the experiments; Wrote the paper; Analyzed and interpreted the data; Contributed reagents, materials, analysis tools or data.

Timothy Ajigepungu Wallah: Conceived and designed the experiments; Wrote the paper; Performed the experiments; Contributed reagents, materials, analysis tools or data.

Benjamin Ahenkorah: Analyzed and interpreted the data; Contributed reagents, materials, analysis tools or data.

Abdallah Yakubu: Performed the experiments; Contributed reagents, materials, analysis tools or data.

## Data availability statement

Data associated with this study has been deposited at https://doi.org/10.6084/m9.figshare.21696584.v1.

## Availability of data and materials

The datasets used and analyzed during the current study are available at https://doi.org/10.6084/m9.figshare.21696584.v1.

## Funding

This research did not receive any specific grant from funding agencies in the public, commercial, or not-for-profit sectors.

## Declaration of competing interest

The authors declare that they have no known competing financial interests or personal relationships that could have appeared to influence the work reported in this paper.
